# Inflammatory related gene IKKα, IKKβ, IKKγ cooperates to determine liver cancer stem cells progression by altering telomere via heterochromatin protein 1-HOTAIR axis

**DOI:** 10.18632/oncotarget.10321

**Published:** 2016-06-29

**Authors:** Jiahui An, Mengying Wu, Xiaoru Xin, Zhuojia Lin, Xiaonan Li, Qidi Zheng, Xin Gui, Tianming Li, Hu Pu, Haiyan Li, Dongdong Lu

**Affiliations:** ^1^ School of Life Science and Technology, Tongji University, Shanghai 200092, China

**Keywords:** IKKα, IKKβ, IKKγ, telomere, HOTAIR

## Abstract

Cancer stem cells are associated with tumor recurrence. IKK is a protein kinase that is composed of IKKα, IKKβ, IKKγ. Herein, we demonstrate that IKKα plus IKKβ promoted and IKKγ inhibited liver cancer stem cell growth in *vitro* and in *vivo*. Mechanistically, IKKα plus IKKβ enhanced and IKKγ inhibited the interplay among HP1α, HP1β and HP1γ that competes for the interaction among HP1α, SUZ12, HEZ2. Therefore, IKKα plus IKKβ inhibited and IKKγ enhanced the activity of H3K27 methyltransferase SUZ12 and EZH2, which methylates H3K27 immediately sites on HOTAIR promoter region. Therefore, IKKα plus IKKβ increased and IKKγ decreased the HOTAIR expression. Strikingly, IKKα plus IKKβ decreases and IKKγ increases the HP1α interplays with DNA methyltransferase DNMT3b, which increases or decreases TERRA promoter DNA methylation. Thus IKKα plus IKKβ reduces and IKKγ increases to recruit TRF1 and RNA polymerase II deposition and elongation on the TERRA promoter locus, which increases or decreases TERRA expression. Furthermore, IKKα plus IKKβ decreases/increases and IKKγ increases/decreases the interplay between TERT and TRRRA/between TERT and TREC. Ultimately, IKKα plus IKKβ increases and IKKγ decreases the telomerase activity. On the other hand, at the telomere locus, IKKα plus IKKβ increases/drcreases and IKKγ decreases/increases TRF2, POT1, pPOT1, Exo1, pExo1, SNM1B, pSNM1B/CST-AAF binding, which keep active telomere regulatory genes and poised for telomere length. Strikingly, HOTAIR is required for IKKα plus IKKβ and IKKγ to control telomerase activity and telomere length. These observations suggest that HOTAIR operates the action of IKKα, IKKβ, IKKγ in liver cancer stem cells. This study provides a novel basis to elucidate the oncogenic action of IKKα, IKKβ, IKKγ and prompts that IKKα, IKKβ, IKKγ cooperate to HOTAR to be used as a novel therapeutic targets for liver cancer.

## INTRODUCTION

IκB kinase (IKK) is composed of three subunits, IKKα, IKKβ, IKKγ, where IKKα and IKKβ are catalytic subunits, and IKKγ is the regulatory subunit. Many diseases are related to IKK [1]. IKKα has been implicated as a key regulator of oncogenesis by affecting NF-ĸB signaling pathways [2]. Activation of IKK-β contributes to cancer pathogenesis and inflammatory disease [3]. IKK inhibition increases drug effectiveness in ovarian cancer [4]. IKK phosphorylation of NF-κB contributes to acquired drug resistance in cancer [5]. Chronic alcohol exposure exacerbates inflammation and triggers pancreatic acinar-to-ductal metaplasia through PI3K/Akt/IKK [7]. IKK phosphorylates RelB to modulate its promoter specificity and promote cell migration downstream of TNF receptors [6]. Moreover, activation of NF-κB requires IKKα and IKKβ kinase activity. Protein kinase C-associated kinase regulates NF-κB activation through inducing IKK activation [7].

Heterochromatin protein1 is involved in chromatin packing and epigenetic gene regulation [8]. The α, β and γ isoforms of HP1 selectively bind to methylated lysine 9 of histone H3 via their chromodomains [9]. Phosphorylation of an HP1-like protein regulates heterochromatin body assembly for DNA elimination [10]. Emerging evidence has shown that HP1α serves a biological role in cancer [11]. HP1 and the histone H3 lys9 methyltransferase Su(var)3-9 is requireed in stem cell self-renewal [12]. Loss of HP1 causes depletion of H3K27me3 and gain of H3K27me2 at constitutive heterochromatin [13]. Furthermore, HP1 regulates this gene's alternative splicing by recruiting splicing factors [14].

Accumulating evidence indicates that HOTAIR plays a critical role in cancer progression. HOTAIR could promote migration and invasion of hepatocellular carcinoma (HCC) cells by inhibiting RBM38 [15]. The Polycomb group (PcG) protein heterodimer EZH2-EED is necessary and sufficient for binding to the lncRNA HOTAIR [16]. Studies suggest that HOTAIR recruits chromatin-modifying complexes to specific target sequences and triggers stemness acquisition [17]. Moreover, Enforced expression of HOTAIR altered histone H3 lysine 27 methylation, gene expression, and increased cancer invasiveness in a manner dependent on Polycomb Repressive Complex 2 (PRC2) [18]. The PRC2 is composed of a trimeric core of SUZ12, EED and EZH1/2, and PRC2 catalyzes histone H3K27 trimethylation (H3K27me3), a hallmark of gene silencing [19]. H3K27 methylations contribute to the role of PRC2 in maintaining cellular growth [20].

TRF2 activity alteration is absorbing and of great concern. Our previous findings suggest that SET1A and CUDR increased TRF2 expression on the transcriptional and translational level through H3K4me3 [21]. The shelterin protein TRF2 is essential for chromosome-end protection. Dyefunction of TRF2 causes chromosome end-to-end fusions, initiating genomic instability. Elevated levels of TRF2 induce telomeric ultrafine anaphase bridges and rapid telomere deletions [22]. Accumulating evidence suggests that the human telomerase reverse transcriptase catalytic subunit (TERT) contributes to cell to elongate telomeres [23]. The telomeric long noncoding RNA Telomeric repeat-containing RNA (TERRA) is important for telomere regulation. TERRA G-quadruplex structure is critical for binding to telomeres [24]. Moreover, TERRA participates in the regulation of telomere length, telomerase activity and heterochromatinization [25, 26]. Resaerches suggest the SNM1B/APOLLO DNA nuclease functions in resolution of replication stress and maintenance of common fragile site stability [27]. CST/AAF, a DNA polα. primase accessory factor, binds POT1b and shortens the extended overhangs produced by Exo1 [28].

In this report, our findings suggest that IKKα plus IKKβ promoted and IKKγ inhibited liver cancer stem cell growth in *vitro* and in *vivo*. Mechanistically, IKKα plus IKKβ enhanced and IKKγ inhibited the interplay among HP1α, HP1β and HP1γ. Therefore, IKKα plus IKKβ increased and IKKγ decreased the TOTAIR expression. Ultimately, IKKα plus IKKβ increases and IKKγ decreases the telomere length and telomerase activity. Of significance, HOTAIR is required for IKKα plus IKKβ and IKKγ to control telomere. This study provides a novel basis to elucidate the oncogenic action of IKKα, IKKβ, IKKγ.

## RESULTS

### IKKα, IKKβ, IKKγ influence on liver cancer stem cells growth *in vitro*

**Figure 1:**
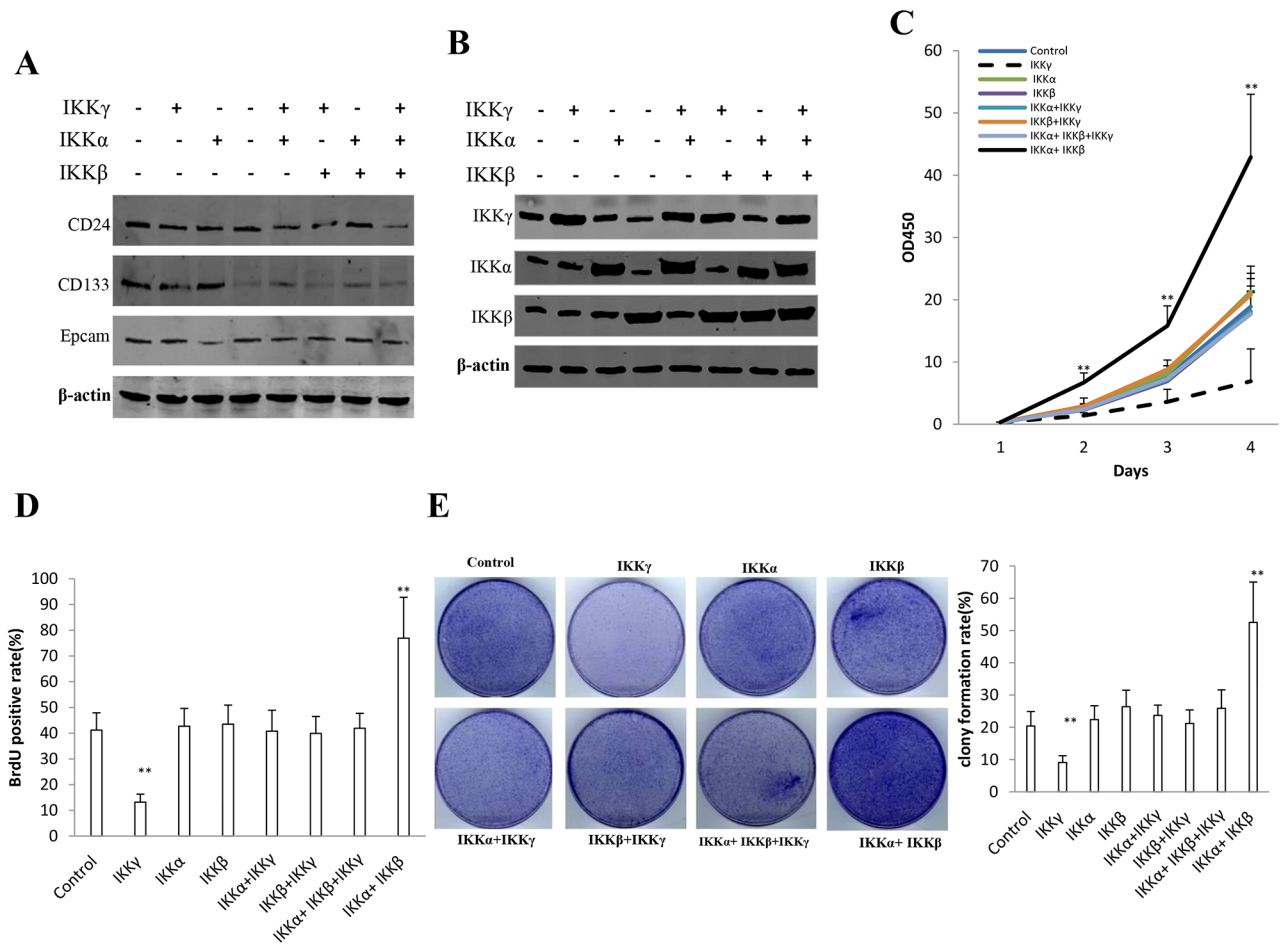
IKKα, IKKβ, IKKγ influence on human liver cancer stem cells (hLCSC) growth *in vitro* **A.** The Western blotting analysis of CD133, CD24, Epcam in stable hLCSC cell lines transfected with pcDNA3.1-IKKα, pcDNA3.1-IKKβ, pcDNA3.1-IKKγ respectively.β-actin as internal control. **B.** The Western blotting analysis of IKKα, IKKβ, IKKγ in stable hLCSC cell lines transfected with pcDNA3.1-IKKα, pcDNA3.1-IKKβ, pcDNA3.1-IKKγ respectively.β-actin as internal control. **C.** Cells growth assay using CCK8. Each value was presented as mean±standard error of the mean (SEM). Data are means of value from three independent experiments, bar±SEM. **, P<0.01; *, P<0.05. **D.** S phase cells assay using BrdU. Each value was presented as mean±standard error of the mean (SEM). Data are means of value from three independent experiments, bar±SEM. **, P<0.01; *, P<0.05. **E.** Cells soft agar colony formation assay. Each value was presented as mean±standard error of the mean (SEM). Data are means of value from three independent experiments, bar±SEM. **, P<0.01; *, P<0.05.

To address whether synergy of IKKα, IKKβ, IKKγ altered the liver cancer stem cells growth in vitro, we first constructed stable cell lines transfected with plasmid with IKKα, IKKβ, IKKγ. As shown in Figure 1A, the expression of CD24, CD133, Epcam is positive in eight stable cell lines (IKKα, IKKβ, IKKγ, IKKα+IKKβ, IKKβ+IKKγ, IKKα+IKKγ, IKKα+IKKβ+IKKγ). Our results showed that IKKα, IKKβ, IKKγ expression were increased in the senen stable cell lines (IKKα, IKKβ, IKKγ, IKKα+IKKβ, IKKβ+IKKγ, IKKα+IKKγ, IKKα+IKKβ+IKKγ) than in control group respectively (Figure 1B). IKKα plus IKKβ overexpression accelerated the growth of liver cancer stem cells to a significantly greater extent when compared with the other cells (P<0.05 or 0.01), as well as single IKKγ overexpression inhibited the growth of liver cancer stem cells compared with the other cells (P<0.05 or 0.01) (Figure 1C). In BrdU staining (a S phase cells assay), the BrdU positive cells rates were 41.2±6.7%,42.7±6.9%, 43.5±7.4%,40.8±8.1%,39.9±6.6%,41.9±5.8% in control, IKKα, IKKβ, IKKβ+IKKγ, IKKα+IKKγ, IKKα+IKKβ+IKKγ group respectively. However, the BrdU positive cells rates was 76.9±15.9% in the IKKα plus IKKβ overexpressed group (p<0.01), while only 13.2±3.1% BrdU positive cells rates was in the single IKKγ overexpressed group (p<0.01) (Figure 1D). In colony-formation efficiency assay, the colonies formed-rates were 20.4±4.5%,22.4±4.3%, 26.4±5.1%,23.7±3.2%,21.2±4.2%,25.9±5.7% in control, IKKα, IKKβ, IKKβ+IKKγ, IKKα+IKKγ, IKKα+IKKβ+IKKγ group respectively. However, the colonies formed-rate was 52.5±12.5% in the IKKα plus IKKβ overexpressed group (p<0.01), while only 9.1±2.1% colonies formed-rate was in the single IKKγ overexpressed group (p<0.01) (Figure 1E). Taken together, IKKα plus IKKβ promoted and IKKγ inhibited liver cancer stem cell growth *in vitro*.

### IKKα, IKKβ, IKKγ influence on liver cancer stem cells growth *in vivo*

**Figure 2:**
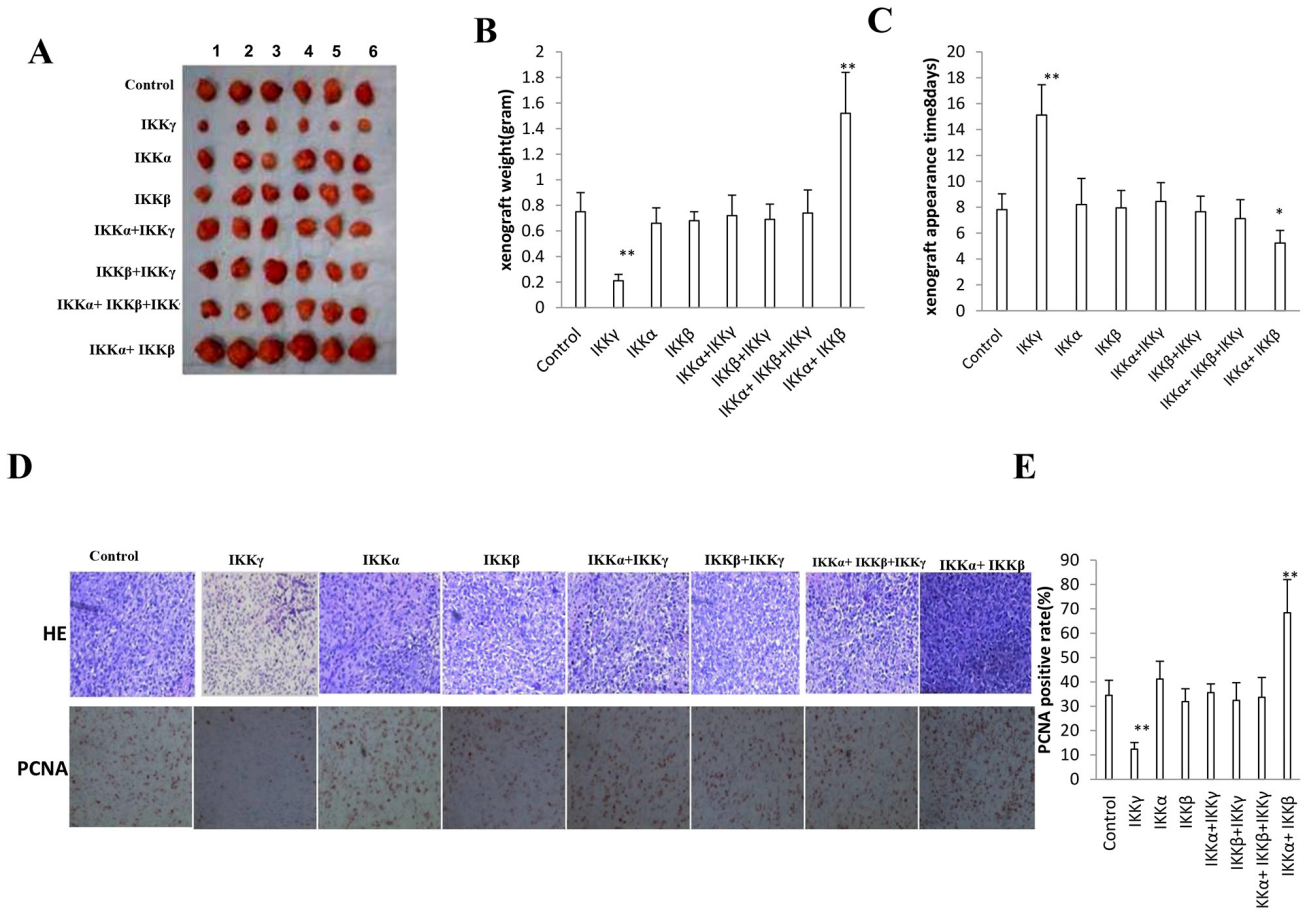
IKKα, IKKβ, IKKγ influence on liver cancer stem cells growth *in vivo* **A.** The mice were stratified and the tumors were recovered. The photography of xenograft tumor in the eight groups (indicated in left). **B.** The wet weight of each tumor was determined for each mouse. Each value was presented as mean±standard error of the mean (SEM). bar±SEM. **, P<0.01; *, P<0.05. **C.** The Xenograft appearance time (days). Each value was presented as mean±standard error of the mean (SEM). bar±SEM. **, P<0.01; *, P<0.05. **D.** A portion of each tumor was fixed in 4% paraformaldehyde and embedded in paraffin for histological hematoxylin-eosin (HE) staining (*upper*) and anti-PCNA immunostainning (*lower*). (original magnification×100). **E.** PCNA positive cells analysis. Each value was presented as mean±standard error of the mean (SEM). bar±SEM. **, P<0.01; *, P<0.05.

To determine whether IKKα, IKKβ, IKKγ, IKKα+IKKβ, IKKβ+IKKγ, IKKα+IKKγ, IKKα+IKKβ+IKKγ could affect the tumorigenesis in vivo, the aforementioned eight stable cell lines were injected subcutaneously at armpit into Balb/C mice. As Expected, significant differences in tumor weights were observed among some groups. As shown in Figure 2A, 2B, when IKKα and IKKβ were co-overexpressed, the tumor weight increased significantly compared to the control, IKKα, IKKβ, IKKβ+IKKγ, IKKα+IKKγ, IKKα+IKKβ+IKKγ overexpression group (1.52±0.32 gram vs 0.75±0.15 gram, 0.66±0.12 gram, 0.68±0.07 gram, 0.72±0.16 gram, 0.69±0.12 gram, 0.74±0.18 gram, p<0.01, respectively). Conversely, when single IKKγ was only overexpressed, the tumor weight significantly compared to the control, IKKα, IKKβ, IKKβ+IKKγ, IKKα+IKKγ, IKKα+IKKβ+IKKγ overexpression group (0.21±0.05 gram vs 0.75±0.15 gram, 0.66±0.12 gram, 0.68±0.07 gram, 0.72±0.16 gram, 0.69±0.12 gram, 0.74±0.18 gram, p<0.01, respectively), and there, however, were no significant difference among control, IKKα, IKKβ, IKKβ+IKKγ, IKKα+IKKγ, IKKα+IKKβ+IKKγ overexpression cell lines (P>0.05). We also found early tumor formation in the IKKα and IKKβ co-overexpressed group (5.23±0.98 days) and later tumor formation in single IKKγ overexpressed group (15.12±2.34 days) compared to the control, IKKα, IKKβ, IKKβ+IKKγ, IKKα+IKKγ, IKKα+IKKβ+IKKγ overexpression group (7.81±1.23 days, 8.21±2.01 days, 7.95±1.34 days, 8.45±1.45 days, 7.65±1.21 days, 7.12±1.46 days, p<0.01, respectively) (Figure 2C). Pathological picture (HE stain) of xenograft tumor showed that tumor tissue possessed more poor-differentiation cells and less moderately or well-differentiation cells in the IKKα and IKKβ co-overexpressed group, and less poor-differentiation cells and more moderately or well-differentiation cells in single IKKγ overexpressed group than that of control, IKKα, IKKβ, IKKβ+IKKγ, IKKα+IKKγ, IKKα+IKKβ+IKKγ overexpressed groups (Figure 2D, Upper). As shown in Figure 2D lower & Figure 2E, the number of PCNA positive cells is higher in IKKα and IKKβ co-overexpressed group (68.4±13.6%) and lower in single IKKγ overexpression group (12.4±2.7) compared to the control, IKKα, IKKβ, IKKβ+IKKγ, IKKα+IKKγ, IKKα+IKKβ+IKKγ overexpressed groups (34.5±6.2%,41.2±7.3%,31.9±5.3%,35.6±3.6%,32.4±7.4%,33.1±8.1%, p<0.01, respectively), and there, however, were no significant difference among control, IKKα, IKKβ, IKKβ+IKKγ, IKKα+IKKγ, IKKα+IKKβ+IKKγ overexpressed groups (P>0.05). Taken together, these observations suggest IKKα plus IKKβ promoted and single IKKγ inhibited liver cancer stem cell growth *in vivo*.

### IKKα, IKKβ, IKKγ alter the methylation of HistoneH3 on lysine 27 dependent on HP1

**Figure 3:**
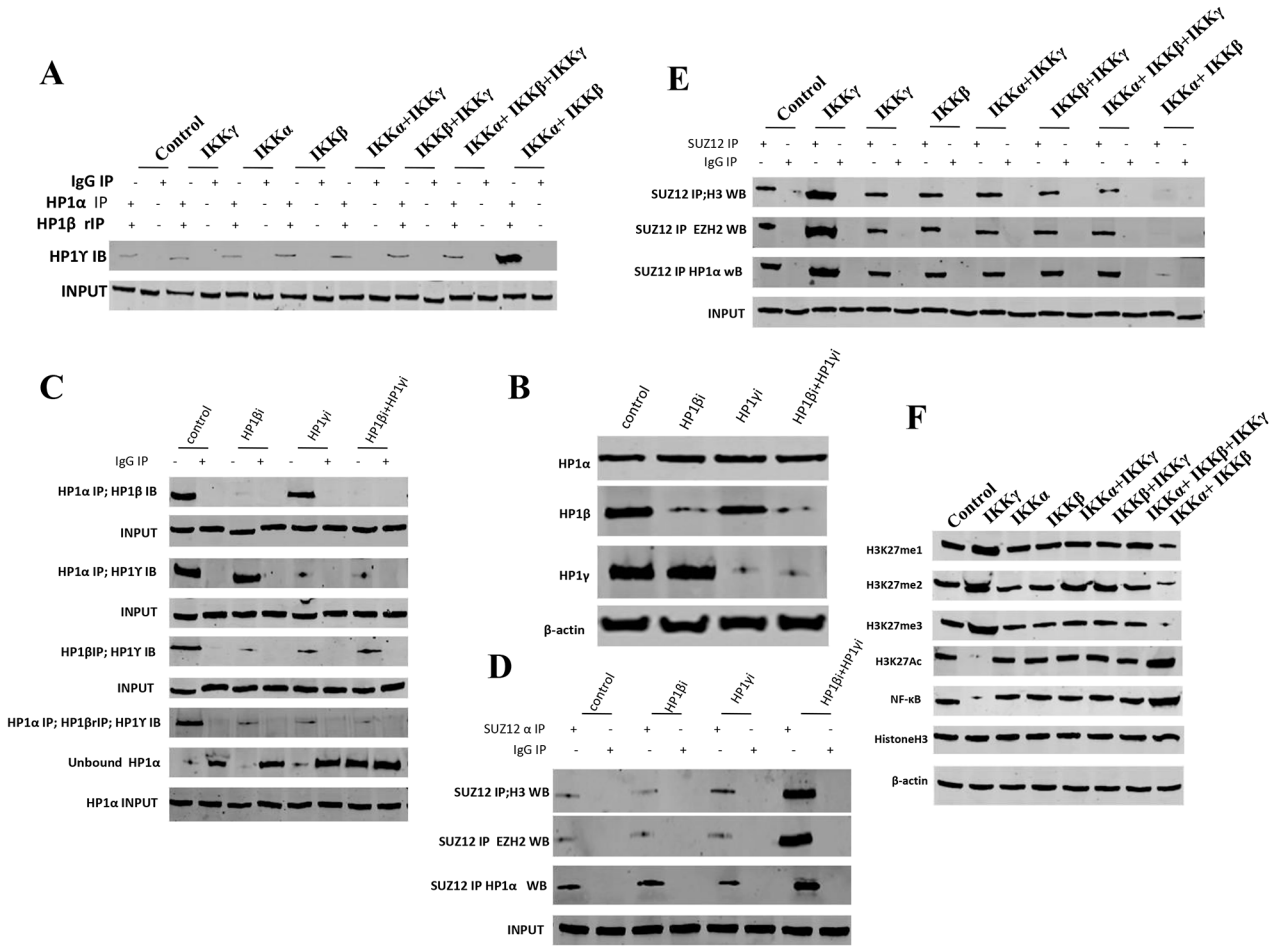
IKKα, IKKβ, IKKγ alter the methylation of HistoneH3 on lysine 27 dependent on HP1 **A.** Repeat Co-Immunoprecipitation (IP) with anti-HP1α, anti-HP1β followed by western blotting with, anti-HP1γ in liver cancer stem cells transfected with pcDNA3.1-IKKα, pcDNA3.1-IKKβ, pcDNA3.1-IKKγ, respectively. IgG IP as negative control. Western blotting with, anti-HP1γ as INPUT. **B.** Western blotting with anti-HP1α, anti-HP1β, anti-HP1γ in liver cancer stem cells transfected with pGFP-V-RS-HP1β or pGFP-V-RS-HP1γ, respectively. β-actin as internal control. **C.** Co-Immunoprecipitation (IP) with anti-HP1α or anti-HP1β followed by western blotting with, anti-HP1β or anti-HP1γ in liver cancer stem cells transfected with pGFP-V-RS-HP1β or pGFP-V-RS-HP1γ, respectively. IgG IP as negative control. Western blotting with anti-HP1β or anti-HP1γ as INPUT. **D.** Co-Immunoprecipitation (IP) with anti-SUZ12 followed by western blotting with anti-Histone H3, anti-EZH2, anti-HP1 α in liver cancer stem cells transfected with pGFP-V-RS-HP1β or pGFP-V-RS-HP1γ, respectively. IgG IP as negative control. Western blotting with anti-SUZ12 as INPUT. **E.** Co-Immunoprecipitation (IP) with anti-SUZ12 followed by western blotting with anti-Histone H3, anti-EZH2, anti-HP1 α in liver cancer stem cells transfected with pcDNA3.1-IKKα, pcDNA3.1-IKKβ, pcDNA3.1-IKKγ. respectively. IgG IP as negative control. Western blotting with anti-SUZ12 as INPUT. **F.** Western blotting with anti-H3K27me1, anti-H3K27me2, anti-H3K27me3, anti-H3K27Ac, anti-NFκB and anti-H3 in liver cancer stem cells transfected with pcDNA3.1-IKKα, pcDNA3.1-IKKβ, pcDNA3.1-IKKγ, respectively. β-actin as internal control.

To prove whether IKKα, IKKβ, IKKγ influence on the methylation of HistoneH3 on lysine 27, we performed western blotting and IP assay in these groups. As shown in Figure 3A, compared to the control, IKKα, IKKβ, IKKβ+IKKγ, IKKα+IKKγ, IKKα+IKKβ+IKKγ overexpression group, the interplay among HP1α, HP1β and HP1γ was significantly increased when IKKα and IKKβ were co-overexpressed. Conversely, the interplay among HP1α, HP1β and HP1γ was significantly decreased when single IKKγ was overexpressed. Furthermore, when HP1β knockdown, HP1γ knockdown or HP1β plus HP1γ knockdown (Figure 3B), the interplay among HP1α, HP1β and HP1γ was significantly decreased, and the unbound HP1α was significantly increased in HP1β plus HP1γ knockdown group (Figure 3C). Furthermore, the interplay among SUZ12, EZH2, Histone3 and HP1α was significantly increased in HP1β plus HP1γ knockdown group (Figure 3D). Compared to the control, IKKα, IKKβ, IKKβ+IKKγ, IKKα+IKKγ, IKKα+IKKβ+IKKγ overexpression group, the interplay among SUZ12, EZH2, Histone3 and HP1α was significantly decreased when IKKα and IKKβ were co-overexpressed. Conversely, the interplay among SUZ12, EZH2, HistoneH 3 and HP1α was significantly increased when single IKKγ was overexpressed (Figure 3E). Significantly, compared to the control, IKKα, IKKβ, IKKβ+IKKγ, IKKα+IKKγ, IKKα+IKKβ+IKKγ overexpression group, the H3K27me, H3K27me2 and H3K27me3 were significantly decreased when IKKα and IKKβ were co-overexpressed. Conversely, the H3K27me, H3K27me2 and H3K27me3 was significantly increased when single IKKγ was overexpressed, (Figure 3D). Moreover, the H3K27Ac and NF-κB were significantly increased when IKKα and IKKβ were co-overexpressed. Conversely, the H3K27Ac and NF-κB were significantly increased in single IKKγ overexpressing group (Figure 3E). Taken together, IKKα, IKKβ, IKKγ alter the modification of Histone H3 on lysine 27 dependent on HP1.

### IKKα, IKKβ, IKKγ regulate HOTAIR expression dependent on H3K27me3

**Figure 4:**
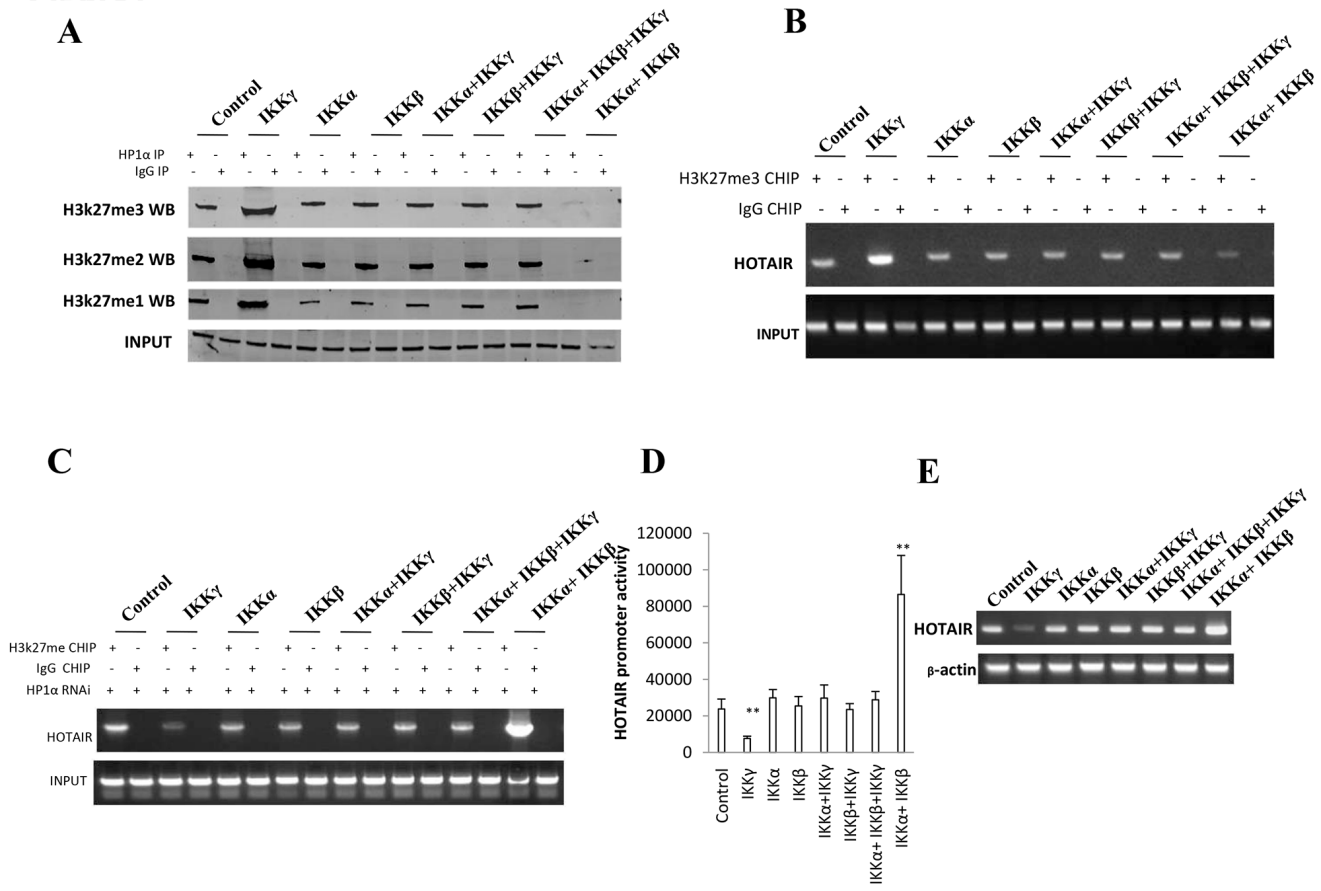
IKKα, IKKβ, IKKγ regulate HOTAIR expression dependent on H3K27me3 **A.** Co-Immunoprecipitation (IP) with anti-HP1α followed by western blotting with, anti-H3K27me1, anti-H3K27me2, anti-H3K27me3 in liver cancer stem cells transfected with pcDNA3.1-IKKα, pcDNA3.1-IKKβ, pcDNA3.1-IKKγ respectively. IgG IP as negative control. Western blotting with anti-HP1 α as INPUT. **B.** Chromatin Immunoprecipitation (CHIP) with anti-H3K27me3 followed by PCR with HOTAIR promoter primers in liver cancer stem cells transfected with pcDNA3.1-IKKα, pcDNA3.1-IKKβ, pcDNA3.1-IKKγ respectively. IgG CHIP as negative control. PCR for HOTAIR promoter as INPUT. **C.** Chromatin Immunoprecipitation (CHIP) with anti-H3K27me3 followed by PCR with HOTAIR promoter primers in HP1 α depleted liver cancer stem cells transfected with pcDNA3.1-IKKα, pcDNA3.1-IKKβ, pcDNA3.1-IKKγ respectively. IgG CHIP as negative control. PCR for HOTAIR promoter as INPUT. **D.** HOTAIR promoter luciferase activity assay in liver cancer stem cells transfected with pcDNA3.1-IKKα, pcDNA3.1-IKKβ, pcDNA3.1-IKKγ, respectively. Data represent mean±SEM, n=3, **p<0.01, *p<0.05 represents difference significane. **E.** RT-PCR with HOTAIR primers in liver cancer stem cells transfected with pcDNA3.1-IKKα, pcDNA3.1-IKKβ, pcDNA3.1-IKKγ respectively. β-actin as internal control.

To investigate whether IKKα, IKKβ, and IKKγ influence on HOTAIR expression, we first performed IP assay in liver cancer stem cells. As shown in Figure 4A, compared to the control, IKKα, IKKβ, IKKβ+IKKγ, IKKα+IKKγ, IKKα+IKKβ+IKKγ overexpression group, when IKKα and IKKβ were co-overexpressed, the interplay between HP1α and H3K27me1/2/3 was significantly decreased. Conversely, when single IKKγ was overexpressed, the interplay between HP1α and H3K27me3 was significantly decreased. Furthermore, the loadeing of H3K27me3 onto the HOTAIR promoter region was significantly decreased when IKKα and IKKβ were co-overexpressed. Conversely, when single IKKγ was overexpressed, the loading of H3K27me3 onto the HOTAIR promoter region was significantly decreased (Figure 4B). However, the action was abolished when HP1α was knocked down in these liver cancer stem cells (Figure 4C). As shown in Figure 4D, compared to the control, IKKα, IKKβ, IKKβ+IKKγ, IKKα+IKKγ, IKKα+IKKβ+IKKγ overexpression group, the HOTAIR promoter luciferase activity was significantly increased when IKKα and IKKβ were co-overexpressed. Conversely, the HOTAIR promoter luciferase activity was significantly decreased when single IKKγ was overexpressed. As shown in Figure 4E, when IKKα and IKKβ were co-overexpressed, the HOTAIR exptression was significantly increased. Conversely, when single IKKγ was overexpressed, the HOTAIR experssion was significantly decreased. Taken together, IKKα, IKKβ, and IKKγ regulate HOTAIR expression dependent on H3K27me3.

### IKKα, IKKβ, and IKKγ control telomerase activity

**Figure 5:**
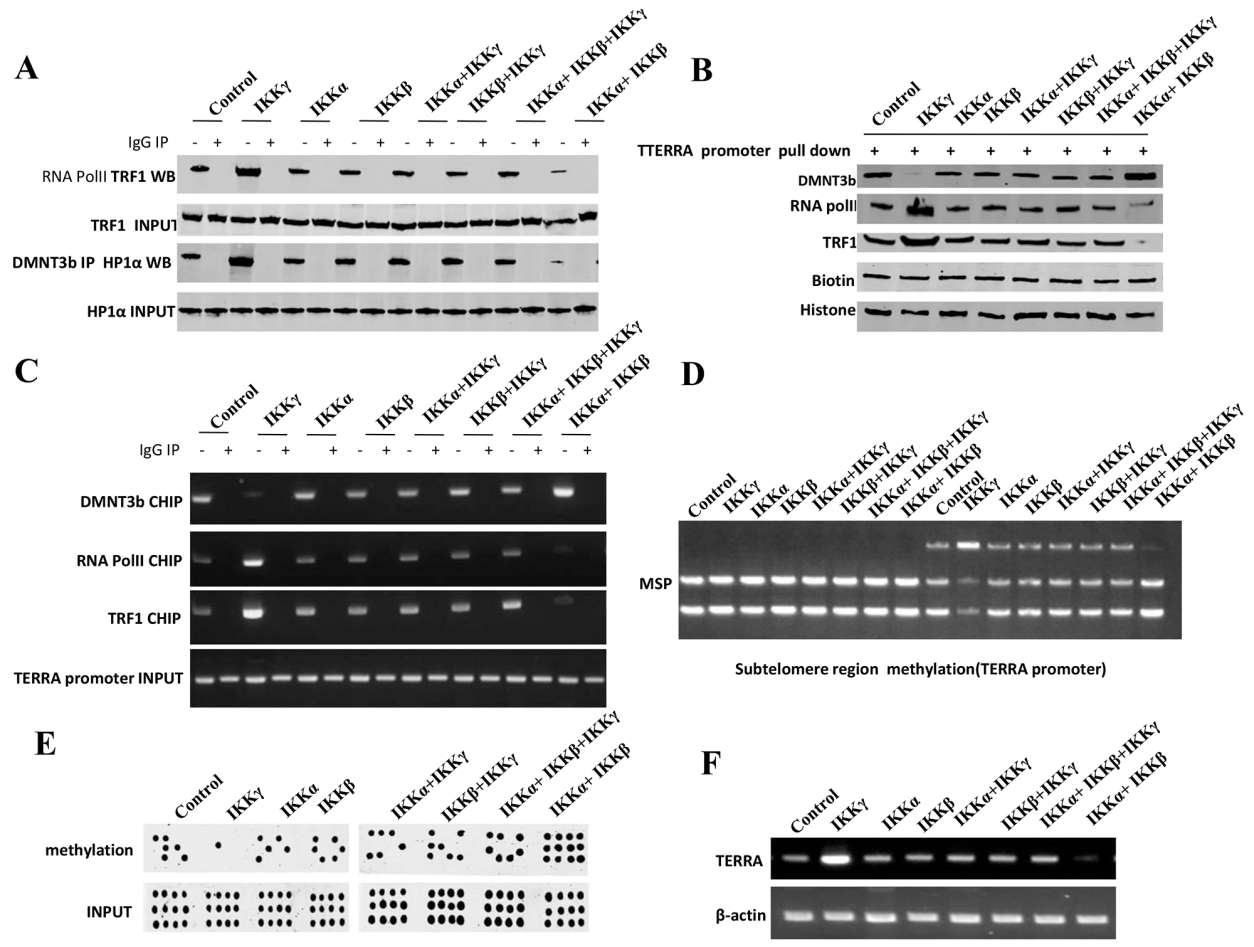
IKKα, IKKβ and IKKγ regulate TERRA expression **A.** Co-Immunoprecipitation (IP) with anti-RNA polII or anti-DNMT3b followed by western blotting with, anti-TRF1 or anti-HP1α in liver cancer stem cells transfected with pcDNA3.1-IKKα, pcDNA3.1-IKKβ, pcDNA3.1-IKKγ respectively. IgG IP as negative control. Western blotting with anti-TRF1 or anti-HP1α as INPUT. **B.** Biotin-TERRA promoter DNA pulldown followed by Western blotting with anti-DNMT3b, anti-TRF1, anti-RNA polII in liver cancer stem cells transfected with pcDNA3.1-IKKα, pcDNA3.1-IKKβ, pcDNA3.1-IKKγ respectively. Biotin as INPUT and Histone as internal control. **C.** Chromatin Immunoprecipitation (CHIP) with anti-DNMT3b, anti-TRF1, anti-RNA polII followed by PCR with TERRA promoter primers in liver cancer stem cells transfected with pcDNA3.1-IKKα, pcDNA3.1-IKKβ, pcDNA3.1-IKKγ respectively. IgG CHIP as negative control. PCR for TERRA promoter as INPUT. **D.** TREEA promoter (Subtelomere region) methylation analysis by MspI plus BamHI digestion in liver cancer stem cells transfected with pcDNA3.1-IKKα, pcDNA3.1-IKKβ, pcDNA3.1-IKKγ respectively. **E.** TERRA promoter methylation analysis by Methylated DNA Immunoprecipitation (MeDIP)-Dot blot-western blotting with anti-5-Methylcytosine (5-mC) in expression in liver cancer stem cells transfected with pcDNA3.1-IKKα, pcDNA3.1-IKKβ, pcDNA3.1-IKKγ respectively. **F.** RT-PCR with TERRA primers in liver cancer stem cells transfected with pcDNA3.1-IKKα, pcDNA3.1-IKKβ, pcDNA3.1-IKKγ respectively. β-actin as internal control.

**Figure 6:**
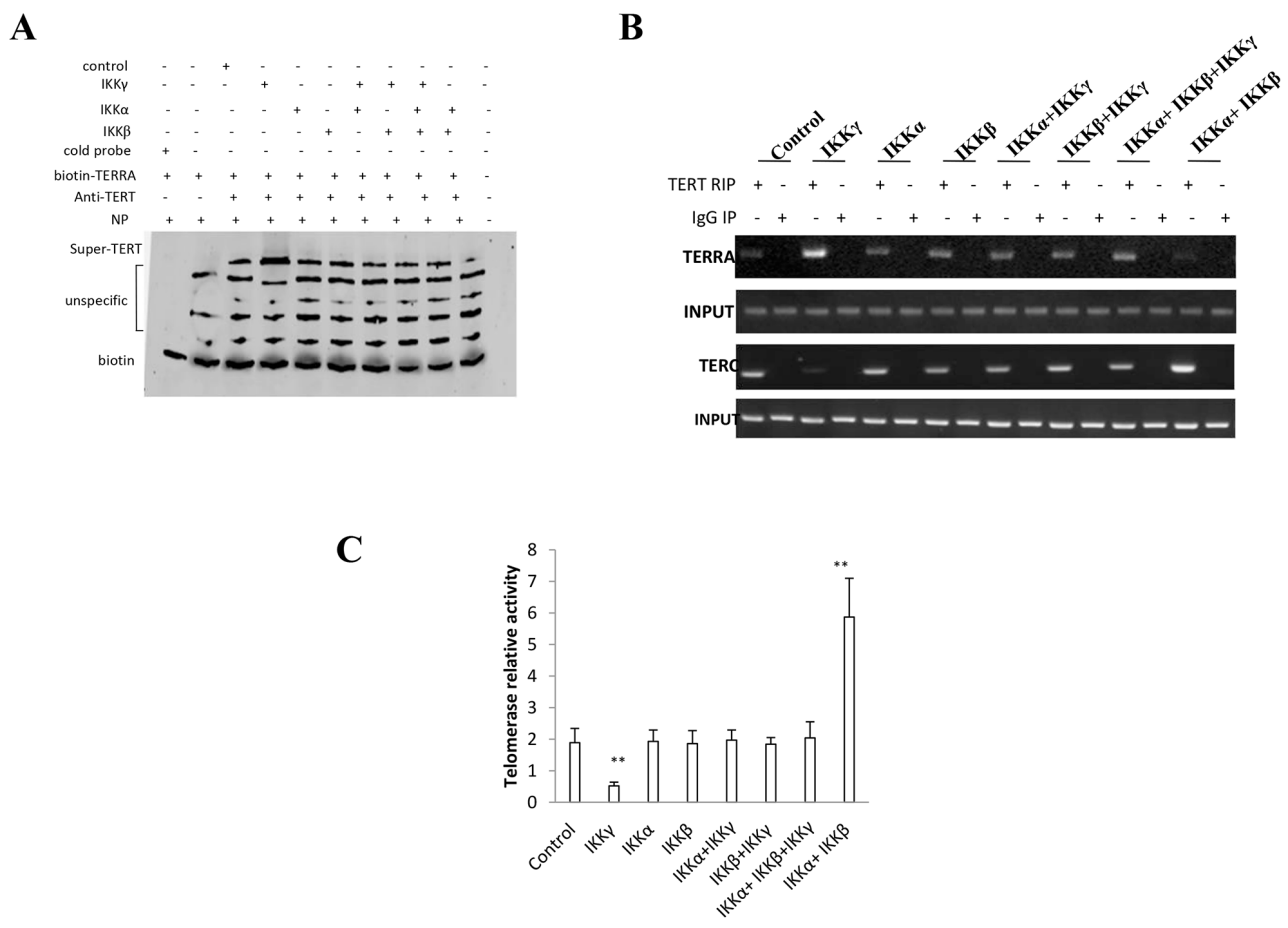
IKKα, IKKβ, IKKγ control telomerase activity **A.** Super-EMSA assay with anti-TERT and biotin-TERRA probe in primers in liver cancer stem cells transfected with pcDNA3.1-IKKα, pcDNA3.1-IKKβ, pcDNA3.1-IKKγ respectively. Biotin as control. **B.** RNA Immunoprecipitation (RIP) with anti-TERT followed by RT-PCR with TERC and TERRA primers in liver cancer stem cells transfected with pcDNA3.1-IKKα, pcDNA3.1-IKKβ, pcDNA3.1-IKKγ respectively. IgG RIP as negative control. RT-PCR for TERC or TERRA as INPUT. **C.** Telomerase activity assay with TRAP method primers in liver cancer stem cells transfected with transfected with pcDNA3.1-IKKα, pcDNA3.1-IKKβ, pcDNA3.1-IKKγ respectively. Each value was presented as mean±standard error of the mean (SEM). **, P<0.01.

To investigate whether IKKα, IKKβ, IKKγ influence on telomere, we first consider to analyse the alteration of telomerase activity. As shown in Figure 5A, compared to the control, IKKα, IKKβ, IKKβ+IKKγ, IKKα+IKKγ, IKKα+IKKβ+IKKγ overexpression group, the interplay between RNAPolIII and TRF1, DNMT3b and HP1α were significantly increased when IKKα and IKKβ were co-overexpressed,. Conversely, the interplay between RNAPolIII and TRF1, DNMT3b and HP1α were significantly decreased when single IKKγ was overexpressed. Moreover, the interplay between TERRA promoter DNA probe and DNMT3b was significantly increased, and the interplay between TERRA promoter DNA probe and RNAPolIII, TRF1 were significantly decreased when IKKα and IKKβ were co-overexpressed. Conversely, when single IKKγ was overexpressed, the interplay between TERRA promoter DNA probe and DNMT3b was significantly decreased, and the interplay between TERRA promoter DNA probe and RNAPolIII, TRF1 were significantly increased (Figure 5B). Furthermore, the loading of DNMT3b on the TERRA promoter region was significantly increased, and the loading of RNAPolIII, TRF1 on the TERRA promoter region were significantly decreased when IKKα and IKKβ were co-overexpressed. Conversely, the loading of DNMT3b on the TERRA promoter region was significantly decreased, and the loading of RNAPolIII, TRF1 on the TERRA promoter region were significantly increased when only IKKγ was overexpressed (Figure 5C). Thereby, the methylation of the TERRA promoter region was significantly increased when IKKα and IKKβ were co-overexpressed. Conversely, the methylation of the TERRA promoter region was significantly decreased when single IKKγ was overexpressed (Figure 5D&5E). Furthermore, the expression of the TERRA promoter region was significantly decreased when IKKα and IKKβ were co-overexpressed. Conversely, the expression of the TERRA promoter region was significantly increased when single IKKγ was overexpressed (Figure 5F). Moreover, compared to the control compared to the control, IKKα, IKKβ, IKKβ+IKKγ, IKKα+IKKγ, IKKα+IKKβ+IKKγ overexpression group, the interplay between TERRA probe and TERT was significantly decreased when IKKα and IKKβ were co-overexpressed,. Conversely, the interplay between TERRA probe and TERT was significantly increased when single IKKγ overexpressed (Figure 6A). The interplay between TERRA and TERT was significantly decreased, and the interplay between TERC and TERT was significantly increased when IKKα and IKKβ were co-overexpressed. Conversely, the interplay between TERRA and TERT was significantly increased, and the interplay between TERC and TERT was significantly decreased when single IKKγ was overexpressed, (Figure 6B). Significantly, the telomerase activity was significantly increased when IKKα and IKKβ were co-overexpressed. Conversely, the telomerase activity was significantly decreased when single IKKγ was overexpressed, (Figure 6C). Taken together, IKKα, IKKβ, and IKKγ control telomerase activity in liver cancer stem cells.

### IKKα, IKKβ, and IKKγ alter telomere length

**Figure 7:**
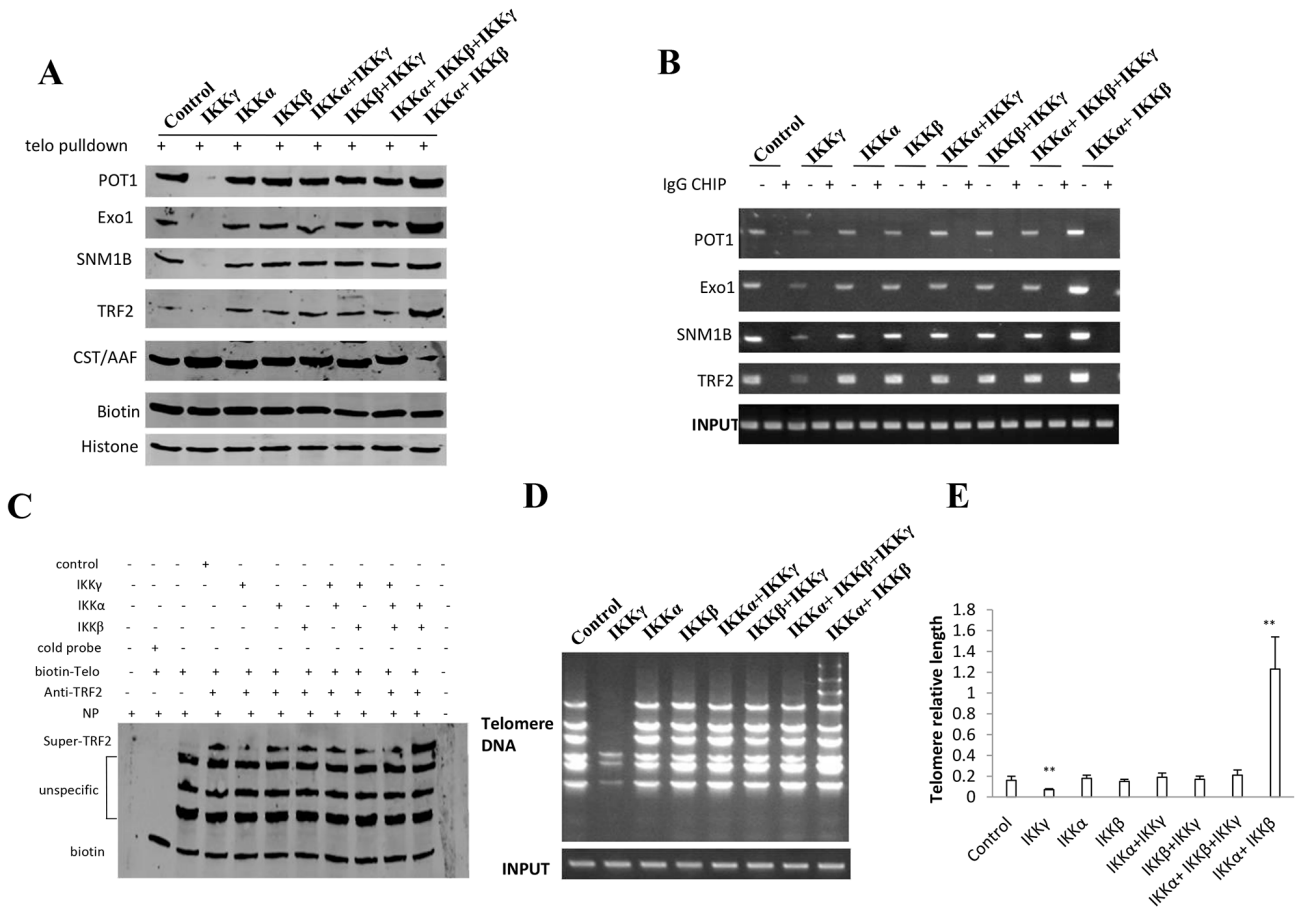
IKKα, IKKβ and IKKγ alter telomere length **A.** Biotin-Telomere DNA pulldown followed by Western blotting with anti-POT1, anti-Exo1, anti-SNM1B, anti-TRF2, anti-CST/AAF in liver cancer stem cells transfected with pcDNA3.1-IKKα, pcDNA3.1-IKKβ, pcDNA3.1-IKKγ respectively. Biotin as INPUT and Histone as internal control. **B.** Chromatin Immunoprecipitation (CHIP) with anti-POT1, anti-Exo1, anti-SNM1B, anti-TRF2 followed by PCR with Telomere DNA primers in liver cancer stem cells transfected with pcDNA3.1-IKKα, pcDNA3.1-IKKβ, pcDNA3.1-IKKγ respectively. IgG CHIP as negative control. PCR for Ttelomere DNA promoter as INPUT. **C.** Super-EMSA assay with anti-TRF2 and biotin-Ttelomere DNA probe in primers in liver cancer stem cells transfected with pcDNA3.1-IKKα, pcDNA3.1-IKKβ, pcDNA3.1-IKKγ respectively. Biotin as control. **D.** The PCR detection of telomere repeat sequence in liver cancer stem cells transfected with pcDNA3.1-IKKα, pcDNA3.1-IKKβ, pcDNA3.1-IKKγ respectively. **E.** The real-time PCR detection of telomere length primers in liver cancer stem cells transfected with pcDNA3.1-IKKα, pcDNA3.1-IKKβ, pcDNA3.1-IKKγ, respectively. Each value was presented as mean±standard error of the mean (SEM). **, P<0.01.

To explore whether IKKα, IKKβ, and IKKγ influence on telomere, we also detected the alteration of telomere length in these liver cancer stem cell. As shown in Figure 7A, compared to the control compared to the control, IKKα, IKKβ, IKKβ+IKKγ, IKKα+IKKγ, IKKα+IKKβ+IKKγ overexpression group, the interplay between the telomere DNA probe and POT1, Exo1, SNM1b, TRF2 were significantly increased, and the interplay between the telomere DNA probe and CST/AAF was significantly decreased when IKKα and IKKβ were co-overexpressed,. Conversely, the interplay between the telomere DNA probe and POT1, Exo1, SNM1b, TRF2 were significantly decreased, and the interplay between the telomere DNA probe and CST/AAF was significantly increased when single IKKγ was overexpressed,. Furthermore, the loading of POT1, Exo1, SNM1b, TRF2 on the telomere DNA were significantly increased when IKKα and IKKβ were co-overexpressed. Conversely, the loading of POT1, Exo1, SNM1b, TRF2 on the telomere DNA were significantly decreased when single IKKγ was overexpressed, (Figure 7B). The interplay between the telomere DNA probe and TRF2 were significantly increased when IKKα and IKKβ were co-overexpressed. Conversely, the interplay between the telomere DNA probe and TRF2 were significantly decreased when single IKKγ was overexpressed, (Figure 7C). Finally, compared to the control, IKKα, IKKβ, IKKβ+IKKγ, IKKα+IKKγ, IKKα+IKKβ+IKKγ overexpression group, the telomere length was significantly increased when IKKα and IKKβ were co-overexpressed. Conversely, the telomere length was significantly decreased when single IKKγ was overexpressed, (Figure 7D&7E). Collectively, these findings suggest that IKKα plus IKKβ increases and IKKγ decreases the telomere length in the liver cancer stem cells.

### HOTAIR depletion blocks function of IKKα, IKKβ, and IKKγ

**Figure 8:**
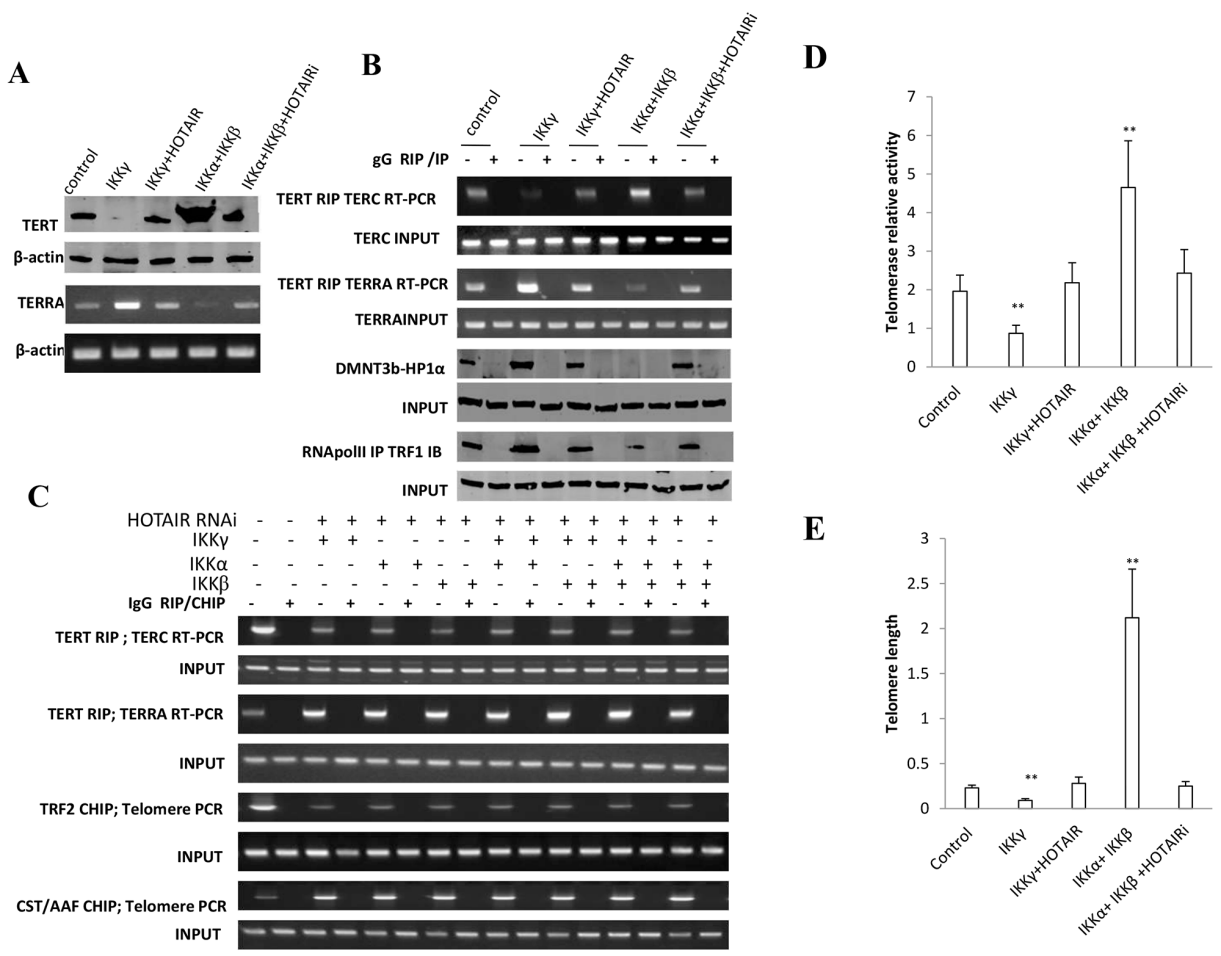
HOTAIR depletion blocks IKKα, IKKβ, IKKγ function on telomere **A.** (***upper***) Western blotting with anti-TERT in liver cancer stem cells transfected with pcDNA3.1, pcDNA3.1-IKKγ, pcDNA3.1-IKKγ plus pCMV6-A-HOTAIR, pcDNA3.1-IKKα plus pcDNA3.1-IKKβ, pcDNA3.1-IKKα plus pcDNA3.1-IKKβ plus pGFP–V-RS-HOTAIR, respectively. β-actin as internal control. (***lower***) RT-PCR with TERRA primers in liver cancer stem cells transfected with pcDNA3.1, pcDNA3.1-IKKγ, pcDNA3.1-IKKγ plus pCMV6-A-HOTAIR, pcDNA3.1-IKKα plus pcDNA3.1-IKKβ, pcDNA3.1-IKKα plus pcDNA3.1-IKKβ plus pGFP–V-RS-HOTAIR, respectively. β-actin as internal control. **B.** (***upper***) RNA Immunoprecipitation (RIP) with anti-TERT followed by RT-PCR with TERC or TERRA promoter primers in liver cancer stem cells transfected with pcDNA3.1, pcDNA3.1-IKKγ, pcDNA3.1-IKKγ plus pCMV6-A-HOTAIR, pcDNA3.1-IKKα plus pcDNA3.1-IKKβ, pcDNA3.1-IKKα plus pcDNA3.1-IKKβ plus pGFP–V-RS-HOTAIR, respectively. IgG RIP as negative control. RT-PCR for TERC or TERRA as INPUT. (***lower***) Co-Immunoprecipitation (IP) with anti-RNA polII or anti-DNMT3b followed by western blotting with, anti-TRF1 or anti-HP1α in liver cancer stem cells transfected with pcDNA3.1, pcDNA3.1-IKKγ, pcDNA3.1-IKKγ plus pCMV6-A-HOTAIR, pcDNA3.1-IKKα plus pcDNA3.1-IKKβ, pcDNA3.1-IKKα plus pcDNA3.1-IKKβ plus pGFP–V-RS-HOTAIR. IgG IP as negative control. Western blotting with anti-TRF1 or anti-HP1α as INPUT. **C.** (***upper***) RNA Immunoprecipitation (RIP) with anti-TERT followed by RT-PCR with TERC and TERRA primers in HOTAIR depleted liver cancer stem cells transfected with pcDNA3.1-IKKα, pcDNA3.1-IKKβ, pcDNA3.1-IKKγ respectively. IgG RIP as negative control. RT-PCR for TERC or TERRA as INPUT. (***lower***) Chromatin Immunoprecipitation (CHIP) with anti-TRF2 or antiCST/AAF followed by PCR with Telomere DNA primers in HOTAIR depleted liver cancer stem cells transfected with pcDNA3.1-IKKα, pcDNA3.1-IKKβ, pcDNA3.1-IKKγ respectively. IgG CHIP as negative control. PCR for Ttelomere DNA promoter as INPUT. **D.** Telomerase activity assay with TRAP method primers in liver cancer stem cells transfected with pcDNA3.1, pcDNA3.1-IKKγ, pcDNA3.1-IKKγ plus pCMV6-A-HOTAIR, pcDNA3.1-IKKα plus pcDNA3.1-IKKβ, pcDNA3.1-IKKα plus pcDNA3.1-IKKβ plus pGFP–V-RS-HOTAIR, respectively. Each value was presented as mean±standard error of the mean (SEM). **, P<0.01. **E.** The real-time PCR detection of telomere length primers in liver cancer stem cells transfected with pcDNA3.1, pcDNA3.1-IKKγ, pcDNA3.1-IKKγ plus pCMV6-A-HOTAIR, pcDNA3.1-IKKα plus pcDNA3.1-IKKβ, pcDNA3.1-IKKα plus pcDNA3.1-IKKβ plus pGFP–V-RS-HOTAIR, respectively. Each value was presented as mean±standard error of the mean (SEM). **, P<0.01.

To determine whether HOTAOR regulated the IKKα, IKKβ, and IKKγ's function on telomeres, we performed rescued-test in liver cancer stem cells. As showed in the Figure 8A, compared to the control, the TERT expression was significantly increased, and the TERRA expression was significantly decreased when IKKα and IKKβ were co-overexpressed. However, the IKKα plus IKKβ function was fully abrogated when HOTAIR was knocked down. Conversely, when single IKKγ was overexpressed, the TERT expression was significantly decreased, and the TERRA expression was significantly dincreased. However, the IKKγ function was fully abrogated when HOTAIR was overexpressed. Furthermore, compared to the control group, when IKKα and IKKβ were co-overexpressed, the interaction between TERT and TERC was significantly increased, and the interaction between TRET andTERRA, DNMT3b and HP1α, RNAPolIII and TRF1 were significantly decreased. However, the IKKα plus IKKβ functions were fully abrogated when HOTAIR was knocked down. Conversely, when single IKKγ was overexpressed, the interaction between TERT and TERC was significantly decreased, and the interaction between TRET and TERRA, DNMT3b and HP1α, RNAPolIII and TRF1 were significantly increased. However, the IKKγ function was fully abrogated when HOTAIR was overexpressed (Figure 8B). Intriguingly, HOTAIR depletion decreased the interplay between TERT and TERC, CST/AAF and telomere, increased the interaction between TERRA and TERT, TRF2 and telomere. However, compared to the IKKα, IKKβ, IKKβ+IKKγ, IKKα+IKKγ, IKKα+IKKβ+IKKγ overexpression group, the interplay between TERT and TERC, TERRA and TERT, TRF2 and telomere, CST/AAF and telomere were significantly not altered when IKKα plus IKKβ or IKKγ were overexpressed in HOTAIR knocked-down liver cancer stem cells (Figure 8C). Ultimately, the telomerase activity and telomere length expression were significantly increased when IKKα and IKKβ were co-overexpressed. However, the IKKα plus IKKβ function was fully abrogated when HOTAIR was knocked down. Conversely, when single IKKγ was overexpressed, the telomerase activity and telomere length expression were significantly decreased. However, the IKKγ function was fully abrogated when HOTAIR was overexpressed (Figure 8D&8E). Taken together, HOTAIR is required for IKKα plus IKKβ and IKKγ to control telomerase activity and telomere length in liver cancer stem cells.

### HOTAIR operates the oncogenic action of IKKα, IKKβ, and IKKγ

**Figure 9:**
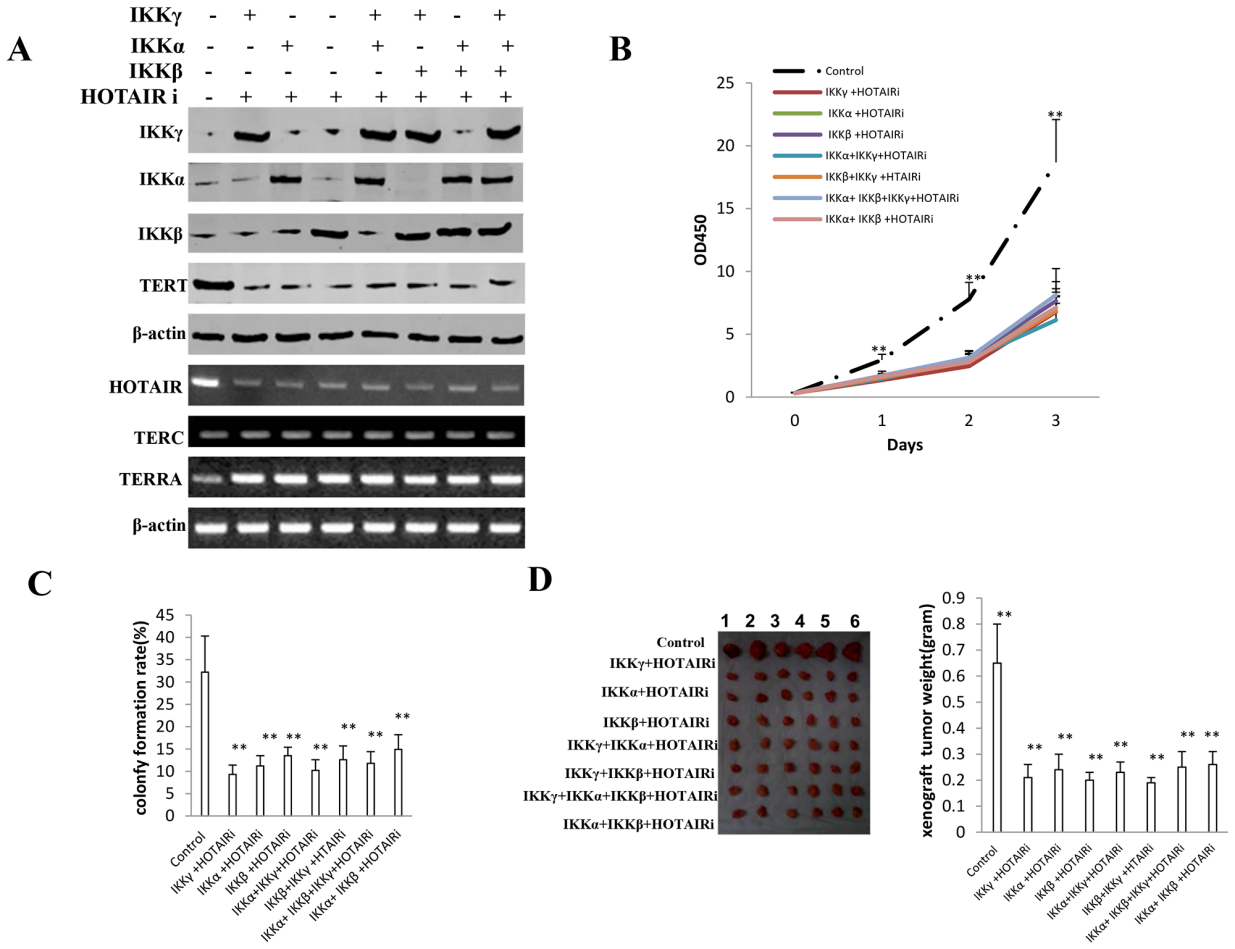
HOTAIR operates the oncogenic action of IKKα, IKKβ, IKKγ **A.** The Western blotting analysis of IKKα, IKKβ, IKKγ, TERT and RT-PCR analysis for HOTAIR, TERC, TERRA in HOTAIR depleted liver cancer stem cell lines transfected with pcDNA3.1-IKKα, pcDNA3.1-IKKβ, pcDNA3.1-IKKγ respectively.β-actin as internal control. **B.** Cells growth assay using CCK8. Each value was presented as mean±standard error of the mean (SEM). Data are means of value from three independent experiments, bar±SEM. **, P<0.01; *, P<0.05. **C.** Cells soft agar colony formation assay. Each value was presented as mean±standard error of the mean (SEM). Data are means of value from three independent experiments, bar±SEM. **, P<0.01; *, P<0.05. **D.** (***left***) The mice were stratified and the tumors were recovered. The photography of xenograft tumor in the eight groups. (***right***) The wet weight of each tumor was determined for each mouse. Each value was presented as mean±standard error of the mean (SEM). bar±SEM. **, P<0.01; *, P<0.05.

To address whether HOTAIR controls the synergy action of IKKα, IKKβ, and IKKγ, we constructed stable cell lines transfected with plasmid with IKKα, IKKβ, IKKγ together with HOTAIR knocked down. As shown in Figure 9A, IKKα, IKKβ, IKKγ expression were increased in the seven stable cell lines (IKKα, IKKβ, IKKγ, IKKα+IKKβ, IKKβ+IKKγ, IKKα+IKKγ, IKKα+IKKβ+IKKγ) than in control group respectively. HOTAIR was knocked down in the stable cell lines (IKKα, IKKβ, IKKγ, IKKα+IKKβ, IKKβ+IKKγ, IKKα+IKKγ, IKKα+IKKβ+IKKγ). TERT expression was reduced and TERRA expression was increased in the HOTAIR knocked down cells. TERC expression was not altered in these cell lines. Although HOTAIR depletion inhibited the cell growth (P<0.01), IKKα plus IKKβ overexpression did not accelerate and IKKγ overexpression did not inhibit the cell growth when compared to the other cells (IKKα, IKKβ, IKKγ, IKKα+IKKβ, IKKβ+IKKγ, IKKα+IKKγ, IKKα+IKKβ+IKKγ) after the HOTAIR was knocked down (P>0.05) (Figure 9B). Although HOTAIR depletion inhibited the cell colony formation ability (32.2±8.1% vs 9.3±2.1%, 11.2±2.3%, 13.5±1.9%, 10.2±2.4%, 12.6±3.1%, 11.8±2.6%, 14.9±3.3%, respectively P<0.01), IKKα plus IKKβ overexpression and IKKγ overexpression did not alter cell colony ability in HOTAIR depleted cells groups (IKKα, IKKβ, IKKγ, IKKα+IKKβ, IKKβ+IKKγ, IKKα+IKKγ, IKKα+IKKβ+IKKγ) (P>0.05) (Figure 9C). Furthermore, although HOTAIR depletion inhibited the xenograft tumor formation (0.65±0.15 gram vs 0.21±0.05 gram, 0.24±0.06 gram, 0.2±0.03 gram, 0.23±0.0 gram, 0.19±0.02 gram, 0.25±0.06 gram,0.26±0.05 gram, respectively, P<0.01), IKKα plus IKKβ overexpression and single IKKγ overexpression did not alter xenograft tumor formation ability in HOTAIR depleted cells groups (IKKα, IKKβ, IKKγ, IKKα+IKKβ, IKKβ+IKKγ, IKKα+IKKγ, IKKα+IKKβ+IKKγ) (P>0.05) (Figure 9D). Taken together, these observations suggest that HOTAIR operates the oncogenic action of IKKα, IKKβ, and IKKγ in liver cancer stem cells.

## DISCUSSION

**Figure 10:**
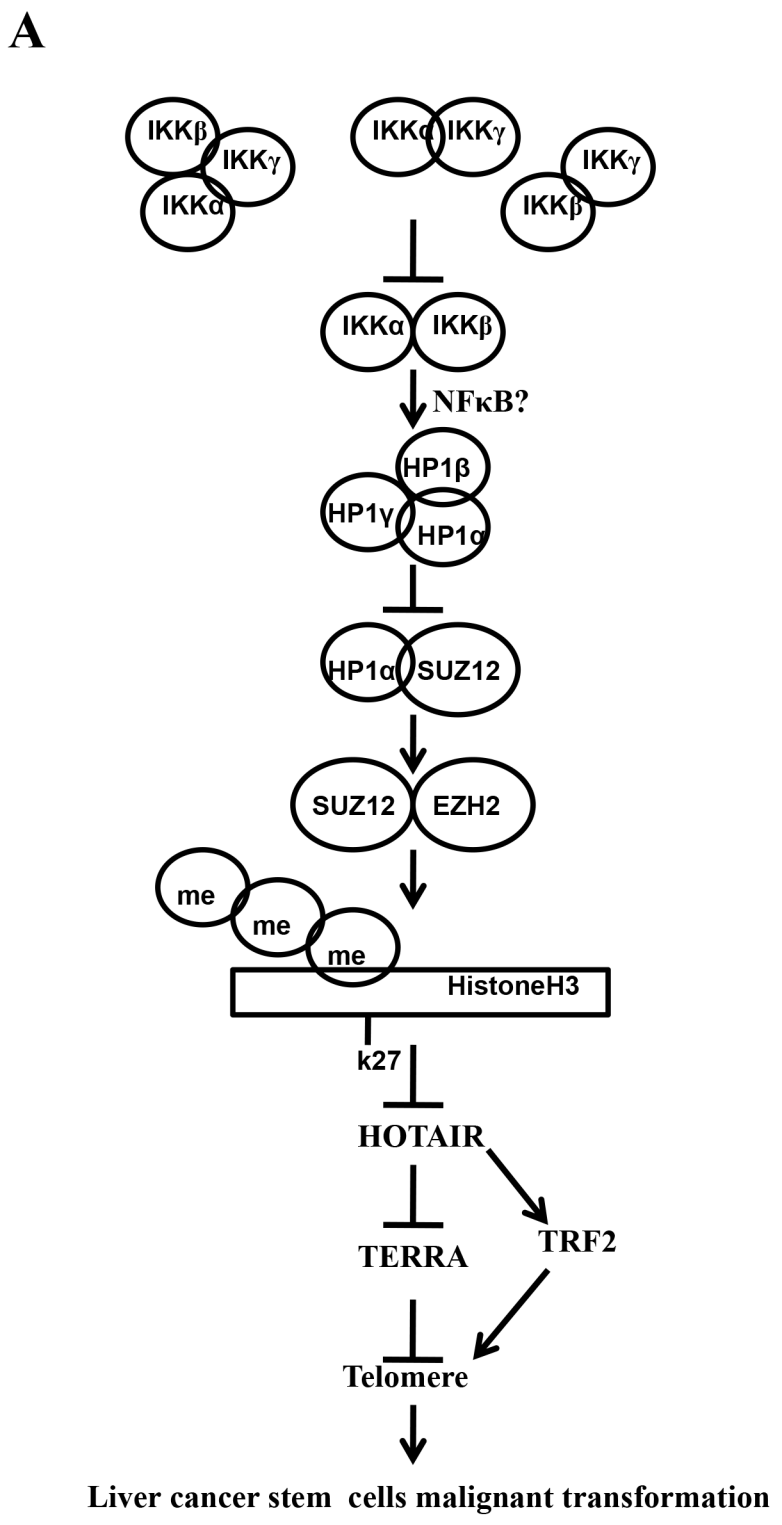
The schematic diagram illustrates a model that that IKKα plus IKKβ promoted and IKKγ inhibited liver cancer stem cell growth *in vitro* and *in vivo* **A.** IKKα plus IKKβ enhanced and IKKγ inhibited the interplay among HP1α, HP1β and HP1γ that competes for the interaction among HP1α, SUZ12, HEZ2. Therefore, IKKα plus IKKβ inhibited and IKKγ enhanced the activity of H3K27 methyltransferase SUZ12 and EZH2, which methylates H3K27 immediately sites on HOTAIR promoter region. Therefore, IKKα plus IKKβ increased and IKKγ decreased the TOTAIR expression. Strikingly, IKKα plus IKKβ decreases and IKKγ increases the HP1α interplays with DNA methyltransferase DNMT3b, which increases or decreases TERRA promoter DNA methylation. So IKKα plus IKKβ reduces and IKKγ increases to recruit TRF1 and RNA polymerase II deposition and elongation on the TERRA promoter locus, which increases or decreases TREEA expression. Further on, IKKα plus IKKβ decreases/increases and IKKγ increases/decrease the interplay between TERT and TRRRA/between TERT and TREC. Ultimately, IKKα plus IKKβ increases and IKKγ decreases the telomerase activity. On the other hand, at the telomere locus, IKKα plus IKKβ increases/drcreases and IKKγ decreases/increases TRF2, POT1, pPOT1, Exo1, pExo1, SNM1B, pSNM1B/CST-AAF binding, which keep active telomere regulatory genes and poised for telomere length. **B.** HOTAIR is required for IKKα plus IKKβ and IKKγ to control telomerase activity and telomere length. HOTAIR depletion blocks the function of IKKα plusIKKβ, IKKγ on telomere.

Cancer stem cells (CSCs) are involved in tumorigenesis. The IKK is critical in cellular proliferation and in tumor-initiating [29]. However, the regulatory mechanism of IKKs has not been elucidated. To our knowledge, this is the first report demonstrating inflammatory related gene IKKα, IKKβ, and IKKγ cooperate to determine liver cancer stem cells progression by altering telomere via heterochromatin protein 1-HOTAIR axis. As shown in Figure 10, we demonstrate that IKKα plus IKKβ promoted and IKKγ inhibited liver cancer stem cell growth in *vitro* and in *vivo*. Mechanistically, IKKα plus IKKβ enhanced and IKKγ inhibited the interplay among HP1α, HP1β and HP1γ that competes for the interaction among HP1α, SUZ12, HEZ2. Therefore, IKKα plus IKKβ inhibited and IKKγ enhanced the activity of H3K27 methyltransferase SUZ12 and EZH2, which methylates H3K27 immediately sites on HOTAIR promoter region. Therefore, IKKα plus IKKβ increased and IKKγ decreased the TOTAIR expression. Strikingly, IKKα plus IKKβ decreases and IKKγ increases the HP1α interplays with DNA methyltransferase DNMT3b, which increases or decreases TERRA promoter DNA methylation. Thus IKKα plus IKKβ reduces and IKKγ increases to recruit TRF1 and RNA polymerase II deposition and elongation on the TERRA promoter locus, which increases or decreases TREEA expression. Furthermore, IKKα plus IKKβ decreases/increases and IKKγ increases/decrease the interplay between TERT and TRRRA/between TERT and TREC. Ultimately, IKKα plus IKKβ increases and IKKγ decreases the telomerase activity. On the other hand, at the telomere locus, IKKα plus IKKβ increases/drcreases and IKKγ decreases/increases TRF2, POT1, pPOT1, Exo1, pExo1, SNM1B, pSNM1B/CST-AAF binding, which keep active telomere regulatory genes and poised for telomere length. Strikingly, HOTAIR is required for IKKα plus IKKβ and IKKγ to control telomerase activity and telomere length. HOTAIR depletion blocks the function of IKKα plus IKKβ, IKKγ on telomere. These observations suggest that HOTAIR operates the action of IKKα, IKKβ, IKKγ in liver cancer stem cells. This study provides a novel basis to elucidate the oncogenic action of IKKα, IKKβ, IKKγ and prompts that IKKα, IKKβ, IKKγ cooperate to HOTAR to be used as a new therapeutic targets for liver cancer.

It is worth mentioning that IKKα, IKKβ, IKKγ may play an important role in hepatocarcinogenesis. In this report, we focused mainly on the view how IKKα, IKKβ, IKKγ function during liver cancer stem cells malignant growth. To this date, accumulating evidence indicates that IKKα, IKKβ, IKKγ influence on cell proliferation. For examples, inhibition of IKK/NF-κB pathway controls stem cell proliferation [30]. IKKβ plays a role during intestinal tumorigenesis [31]. BRAF-induced tumorigenesis is dependent on IKKα [32]. IKKβ could regulates VEGF expression in ovarian cancer as an antiangiogenic target [33]. Our present findings are consistent with some reports.

It is worth noting that our findings in this study provide novel evidence for an active role of IKKα plus IKKβ promotion or IKKγ inhibition of liver cancer stem cell growth. Herein, the involvement of promotion or inhibition of liver cancer stem cells growth based on IKKα, IKKβ, IKKγ is supported by results from two parallel sets of experiments: (1) IKKα plus IKKβ promoted and IKKγ inhibited liver cancer stem cell growth in *vitro*; (2) IKKα plus IKKβ promoted and IKKγ inhibited liver cancer stem cell growth in *vivo*.

Strikingly, our observations suggest that IKKα plus IKKβ increased and IKKγ inhibited HOTAIR expression dependent on tri-methylation of Histone H3 on the twenty-seven lysine. This assertion is based on several observations in IKKα plus IKKβ or IKKγ overexpressed liver cancer stem cells: (1) IKKα plus IKKβ enhanced and IKKγ inhibited the interplay among HP1α, HP1β and HP1γ that competes for the interaction among HP1α, SUZ12, HEZ2. (2) IKKα plus IKKβ inhibited and IKKγ increased methylation of histoneH3 on lysine 27 dependent on the tri-complex of HP1. (3) IKKα plus IKKβ increased and IKKγ decreased the H3K27Ac and NF-κB through H3K27me3. (4) IKKα plus IKKβ increased and IKKγ decreased HOTAIR expression dependent on H3K27me3. Researches indicated heterochromatin causes epigenetic repression that control gene expression and function [34]. HP1α is an essential protein critical for heterochromatin assembly and regulation [35]. Strikingly, HP1 promotes tumor suppressor BRCA1 functions during the DNA damage response [36]. The trimethylation of histone H3 lysine 27 (H3K27me3) contributes to gene repression [37]. NF-κB is involved in inflammation and tumor growth [38].

On the other hand, we find that IKKα, IKKβ, and IKKγ control telomerase activity and telomere length. The stability of telomeres depends upon TRF2, which prevents inappropriate repair [39]. Upon telomere shortening or telomere uncapping induced by loss of TRF2, telomeres elicit a DNA-damage response [40]. Our previous study shows CUDR promotes liver cancer stem cell growth through upregulating TERT [41]. In addition, telomeres are protected from hyper-resection through the repression of the ATM and ATR kinases by TRF2 and TPP1-bound POT1a/b, respectively [42]. Shelterin can protect chromosome ends as a TRF2-tethered TIN2/TPP1/POT1 complex [43]. This assertion is based on several observations in TLR4 overexpression or knockdown liver cancer stem cells: (1) When IKKα and IKKβ were co-overexpressed, the telomerase activity and telomere length were significantly increased. (2) Conversely, when only IKKγ was overexpressed, the telomerase activity and telomere length were significantly decreased. (3) IKKα plus IKKβ decreased and IKKγ increased the HP1α interplay with DNA methyltransferase DNMT3b, which increased or decreased TERRA promoter DNA methylation. (2) IKKα plus IKKβ reduced and IKKγ increased to recruit TRF1 and RNA polymerase II deposition and elongation on the TERRA promoter locus, which increased or decreased TREEA expression. (3) IKKα plus IKKβ decreases/increases and IKKγ increases/decrease the interplay between TERT and TRRRA/between TERT and TREC. (4) IKKα plus IKKβ increases/drcreases and IKKγ decreases/increases TRF2, POT1, pPOT1, Exo1, pExo1, SNM1B, pSNM1B/CST-AAF binding, which keep active telomere regulatory genes and poised for telomere length.

Significantly, HOTAIR is required for IKKα plus IKKβ and IKKγ to control telomerase activity, telomere length and tumorigenesis. HOTAIR is associated with a variety of human cancers, such as breast, liver and endometrial carcinoma [44]. HOTAIR induced colony formation and orthotopic tumor growth [45]. HOTAIR directly decreased WIF-1 expression by promoting its histone H3K27 methylation and then activated the Wnt/β-catenin signaling pathway [46]. Furthermore, HOTAIR promotes proliferation of ovarian cancer cells through regulating PIK3R3 [47]. Herein, the involvement of promotion or inhibition of telomere length or telomerase activity based on IKKα, IKKβ, IKKγ is supported by results from three parallel sets of experiments: (1) HOTAIR depletion abolished the function of IKKα plus IKKβ, IKKγ on telomere length. (2) HOTAIR depletion abolished the function of IKKα plus IKKβ, IKKγ on telomerase. (2) HOTAIR depletion abrogated IKKα plus IKKβ oncogenic function.

Especially, our results showed IKKα plus IKKβ enhanced the interplay among HP1α, HP1β and HP1γ by an unknown mechanism, and then inhibited the activity of H3K27 methyltransferase SUZ12 and EZH2 and increased the HOTAIR expression. However, single IKKα or IKKβ overexpression could not alter HOTAIR expression. Because of excessive HOTAIR, IKKα plus IKKβ increases the interplay between TERT and TREC, the telomerase activity and telomere length. However, single IKKα or IKKβ overexpression could not alter the telomerase activity, increases telomere length. Moreover, because of the increase of telomerase activity and telomere length, IKKα plus IKKβ simultaneous overexpression significantly increased liver cancer stem cells's growth, while single IKKα or IKKβ overexpression could not promote liver cancer stem cells's growth.

In addition, our results showed that HOTAIR depletion blocks IKKα plus IKKβ, IKKγ function on telomere. It suggest HOTAIR is required for IKKα plus IKKβ and IKKγ to control telomerase activity and telomere length. In this study, we found IKKα plus IKKβ enhanced and HP1γ decreased the interplay among HP1α, HP1β and HP1γ by an unknown mechanism, and then IKKα plus IKKβ inhibited and HP1γ enhanced the activity of H3K27 methyltransferase SUZ12 and EZH2. Ultimately, IKKα plus IKKβ increased and HP1γ decreased the HOTAIR expression. Because of HOTAIR, IKKα plus IKKβ increases and HP1γ decreased the interplay between TERT and TREC, the telomerase activity and telomere length. IKK is also a crucial protein kinase that activates NF-κB translocating from cytoplasm to nucleus for DNA binding. Especially, IKK phosphorylates NF-κB to modulate its promoter specificity and promote genes expression. NF-κB could regulates and controls telomere related genes. Although IKK could regulate NF-κB and HOTAIR, both abnormal NF-κB and HOTAIR could alter telomeres through different pathways.

We should explore the function of IKKα plus IKKβ, IKKγ in liver cancer stem cells. For example, what causes strong oncogenic action of IKKα plus IKKβ and triggers IKKγ's actions? How does IKKα plus IKKβ, IKKγ cooperates with HOTAIR? Does IKKα plus IKKβ, IKKγ regulates a series of molecular events liver stem cells malignant growth? Answering these questions will help understand the mechanism about liver stem cell malignant differentiation. In summary, our present data indicated that IKKα plus IKKβ, and IKKγ promotes/inhibits liver cancer stem cells malignant progression through altering telomere length and telomerase activity dependent on HOTAIR, with clinic implications. These observations provide insight into a novel link between IKKα, IKKβ, IKKγ and liver cancer stem malignant transformation.

## MATERIALS AND METHODS

### Human liver cancer stem cell line (hLCSC) sorting

CD133/CD44/CD24/EpCAM MicroBead Kits were purchased from Miltenyi technic (Boston, USA) and MACS® Technology operation according to and the operation according to the manufacturer. In brief, centrifuge cell suspension at 300×g for 10 minutes and. Resuspend cell pellet in 300 μL of buffer per 10^8^ total cells after aspirating supernatant completely. Add 100 μL of FcR Blocking Reagent per 10^8^ total cells and 100 μL of CD133/CD44/CD24/EpCAM MicroBeads per 10^8^ total cells. Mix well and incubate for 30 minutes in the refrigerator (2−8 °C). Wash cells by adding 1−2 mL of buffer per 10^8^ cells and centrifuge at 300×g for 10 minutes. Resuspend up to 10^8^ cells in 500 μL of buffer. Choose an appropriate MACS Column and MACS Separator according to the number of total cells and the number of CD133+/CD44+/CD24+/EpCAM+ cells.

### Cell lines and plasmids

Liver cancer stem cell lines were maintained in Dulbecco's modified Eagle medium (Gibco BRL Life Technologies) supplemented with 10% heat-inactivated fetal bovine serum (Gibco BRL Life Technologies) in a humidified atmosphere of 5% CO_2_ incubator at 37°C. pCMV6-A-GFP, pGFP-V-RS were purchased from Origene (Rockville, MD, USA). pcDNA3.1-IKKα, pcDNA3.1-IKKβ, pcDNA3.1-IKKγ were purchase from Addgene (Cambridge MA, USA). pCMV6-A-GFP-HOTAIR, pGFP-V-RS-HOTAIR, pGFP-V-RS-HP1α, pGFP-V-RS–HP1β, pGFP-V-RS–HP1γ, pGL4-HOTAIR/promoter were prepared by ourselves.

### Cell transfection and stable cell lines cell transfection and stable cell lines

Cells were transfected with DNA plasmids using transfast transfection reagent lipofectamine^R^ 2000 (Invitrogen) according to manufacturer's instructions.

### RT-PCR

Total RNA was purified using Trizol (Invitrogen) according to manufacturer's instructions. cDNA was prepared by using oligonucleotide (dT)_17-18_, random primers, and a SuperScript First-Strand Synthesis System (Invitrogen). PCR analysis was performed under the specical conditions. β-actin was used as an internal control.

### Western blotting

The logarithmically growing cells were washed twice with ice-cold phosphate-buffered saline (PBS, Hyclone) and lysed in a RIPA lysis buffer. Cells lysates were centrifuged at 12,000g for 20 minutes at 4°C after sonication on ice, and the supernatant were separated. After being boiled for 5-10 minutes in the presence of 2-mercaptoethanol, samples containing cells proteins were separated on a 10% sodium dodecyl sulfate-polyacrylamide gel electrophoresis (SDS-PAGE) and transferred onto a nitrocellulose membranes (Invitrogen, Carlsbad, CA, USA). Then blocked in 10% dry milk-TBST (20mM Tris-HCl [PH 7.6], 127mM NaCl, 0.1% Tween 20) for 1 h at 37°C. Following three washes in Tris-HCl pH 7.5 with 0.1% Tween 20, the blots were incubated with 0.2 μg/ml of antibody (appropriate dilution) overnight at 4°C. Following three washes, membranes were then incubated with secondary antibody for 60 min at 37°C or 4°C overnight in TBST. Signals were visualized by ODYSSEY infrared imaging system (LI-COR, Lincoln, Nebraska USA). IRDye 680LT secondary antibodies were purchased from LI-COR scientific company.

### Co-immunoprecipitation (IP)

Cells were lysed in 1 ml of the whole-cell extract buffer A (50mM pH7.6 Tris-HCl, 150mMNaCl, 1%NP40, 0.1mMEDTA, 1.0mM DTT, 0.2mMPMSF, 0.1mM Pepstatine, 0.1mM Leupeptine, 0.1mM Aproine). Five-hundred-microliter cell lysates was used in immunoprecipitation with antibody. In brief, protein was pre-cleared with 30μl protein G/A-plus agarose beads (Santa Cruz, Biotechnology, Inc. CA) for 1 hour at 4°C and the supernatant was obtained after centrifugation (5,000rpm) at 4°C. Precleared homogenates (supernatant) were incubated with 2 μg of antibody and/or normal mouse/rabbit IgG by rotation for 4 hours at 4°C, and then the immunoprecipitates were incubated with 30μl protein G/A-plus agarose beads by rotation overnight at 4°C, and then centrifuged at 5000rpm for 5 min at 4°C. The precipitates were washed five times×10min with beads wash solution (50 mM pH7.6 TrisCl, 150mMNaCl, 0.1%NP-40, 1mM EDTA) and then resuspended in 60μl 2×SDS-PAGE sample loading buffer to incubate for 10 min at 100°C. Then Western blot was performed with a another related antibody indicated in Western blotting.

### RNA immunoprecipitation (RIP)

Cells were lysed (15 min, 4°C) in 100 mM KCl, 5 mM MgCl_2_, 10 mM HEPES [pH 7.0], 0.5% NP40, 1 mM DTT, 100 units/ml RNase OUT (Invitrogen), 400 μM vanadyl-ribonucleoside complex and protease inhibitors (Roche). The lysates were incubated with specific antibody or normal mouse/rabbit IgG overnight at 4°C, followed that the lysates were incubated with protein A/G-plus agarose beads (Santa Cruz, Biotechnology, Inc. CA) 4 hours at 4°C. Then the beads were subsequently washed four times with 50 mM Tris-HCl (pH 7.0), 150 mM NaCl, 1 mM MgCl_2_, and 0.05% NP-40, and twice after addition of 1M Urea. RNA is isolated from the Immunoprecipitates (IPs) and RT-PCR is performed.

### DNA pull down

Cells were lysed by sonication in HKMG buffer (10 mM HEPES, PH7.9, 100 mM KCl, 5 mM MgCl_2_, 100% glycerol, 1 mM DTT, and 0.5% NP40) containing protease and phosphatase inhibitors for the preparation of nuclear exact. Equal amount of cell nuclear extracts were precleared with Streptavidin-agarose Resin (Thermo) for 1 hours, and then were incubated with 1μg biotinylated double-stranded-oligonucleotides and together with 10μg poly(dI-dC) at 4°C for 24 hours. DNA-bound proteins were collected with the incubation with streptavidin-agarose Resin for 1 hour with gently shaking to prevent precipitation in solution. Following 5 washings of the resin bound complex with 0.5-1.0 ml of binding buffer, the samples were boiled and subjected to SDS-PAGE and Western blot analysis.

### Chromatin immunoprecipitation (CHIP) assay

Cells were cross-linked with 1% (v/v) formaldehyde (Sigma) for 10 min at room temperature and stopped with 125 mm glycine for 5 min. Crossed-linked cells were washed with phosphate-buffered saline, resuspended in lysis buffer, and sonicated for 8-10 min in a SONICS VibraCell to generate DNA fragments with an average size of 500 bp or so. Chromatin extracts were diluted 5-fold with dilution buffer, pre-cleared with Protein-A/G-Sepharose beads, and immunoprecipitated with specific antibody on Protein-A/G-Sepharose beads. After washing, elution and de-cross-linking, the ChIP DNA was detected by PCR.

### Super-EMSA (Gel-shift)

Cells were washed and scraped in ice-cold PBS to prepare nuclei for electrophoretic gel mobility shift assay with the use of the gel shift assay system modified according to the manufacturer's instructions (Promega).

### Quantitative telomerase detection

The telomerase activity was measured by using Quantitative Telomerase Detection Kit (MT3010) according to manufacturer's instructions (US Biomax, Inc).

### Telomere length assay

Telomere length assay using Telo TAGGG PCR ELISApuls kit according to manufacturer's instructions (Roche). A standard curve is established by dilution of known quantities of a synthesised 84 mer oligonucleotide containing only TTAGGG repeats.

### Methylation analysis

mthylated DNA Immunoprecipitation (MeDIP)-Dot blot-western blotting with anti-5-Methylcytosine (5-mC) and ethylation analysis by MspI plus BamHI digestion.

### Cells proliferation CCK8 assay

Cells were synchronized in G0 phase by serum deprivation and then released from growth arrest by reexposure to serum, and then cells were grown in complete medium for assay. The cell proliferation reagent CCK8 is purchased from Roch and the operation according to the manufacturer instruction.

### Soft agar colony formation capacity assay

2 × 10^2^ cells were plated on a 6 well plate containing 0.5% (lower) and 0.35% (upper) double layer soft-agar. The 6 well plates were incubated at 37°C in humidified incubator for 14 days. The cells were fed 1-2 times per week with cell culture media (DMEM). Soft-agar colonies on the 6 well plates were stained with 0.5 ml of 0.05% Crystal Violet for more than 1 hour and the colonies were counted.

### BrdU staining

70-80% confluent cells were cultured for 24 hour before treatment with 10μl BrdU (Roche) for 4 hours. Immunofluorescent staining with an anti-BrdU antibody was performed according to the manufacturer's instructions (Becton Dickinson). BrdU positive cells from ten random chosen fields of at least three independent samples were counted.

### Xenograft transplantation *in vivo*

The Four-weeks athymic Balb/C mouse was injected the liver cancer cells at the armpit area subcutaneously. The mice were observed over 4 weeks, and then sacrificed to recover the tumors. The wet weight of each tumor was determined for each mouse. A portion of each tumor was fixed in 4% paraformaldehyde and embedded in paraffin for histological hematoxylin-eosin (HE) staining. The use of mice for this work was reviewed and approved by the institutional animal care and use committee in accordance with China national institutes of health guidelines.

### Statistical analysis

The significant differences between mean values obtained from at least three independent experiments. Each value was presented as mean±standard error of the mean (SEM) unless otherwise noted, with a minimum of three replicates. Student's t-test was used for comparisons, with P<0.05 considered significant.
